# A peer mentoring program for eating disorders: improved symptomatology and reduced hospital admissions, three years and a pandemic on

**DOI:** 10.1186/s40337-024-01051-7

**Published:** 2024-07-15

**Authors:** Anita Raspovic, Rachael Duck, Andrew Synnot, Belinda Caldwell, Andrea Phillipou, David Castle, Richard Newton, Leah Brennan, Zoe Jenkins, Michelle Cunich, Sarah Maguire, Jane Miskovic-Wheatley

**Affiliations:** 1 Eating Disorders Victoria, Abbotsford, Victoria 3067 Australia; 2 InsideOut Institute for Eating Disorders, The University of Sydney and Sydney Local Health District, Sydney, New South Wales 2006 Australia; 3 School of Allied Health, Human Services and Sport, La Trobe University, Melbourne, Victoria 3086 Australia; 4 MAINSTREAM The Australian Centre for Health System Research and Translation in Eating Disorders, Sydney, New South Wales 2006 Australia; 5 Orygen, Melbourne, Victoria 3052 Australia; 6 Centre for Youth Mental Health, The University of Melbourne, Melbourne, Victoria 3000 Australia; 7 Department of Psychological Sciences, Swinburne University of Technology, Melbourne, Victoria 3182 Australia; 8 Department of Mental Health, St Vincent’s Hospital, Melbourne, Victoria 3065 Australia; 9 Department of Mental Health, Austin Health, Melbourne, Victoria 3084 Australia; 10 University of Tasmania and Tasmanian Centre for Mental Health Service Innovation, Hobart, Tasmania 7000 Australia; 11 Peninsula Mental Health Service, Monash University, Frankston, Victoria 3199 Australia; 12 School of Psychology and Public Health, La Trobe University, Wodonga, Victoria 3689 Australia; 13 Boden Initiative, Charles Perkins Centre, Faculty of Medicine and Health (Central Clinical School), The University of Sydney, Camperdown, New South Wales 2006 Australia; 14 Sydney Health Economics Collaborative, Sydney Local Health District, Camperdown, New South Wales 2050 Australia; 15 Sydney Institute for Women, Children and Their Families, Sydney Local Health District, Camperdown, New South Wales 2050 Australia; 16 Cardiovascular Initiative, Faculty of Medicine and Health, The University of Sydney, Camperdown, New South Wales 2006 Australia; 17 Faculty of Medicine and Health (Central Clinical School), The University of Sydney, Sydney, New South Wales 2050 Australia

**Keywords:** Peer mentoring, Peer work, Lived experience, Eating disorders, Treatment, Intervention, Hospitalisations, Implementation

## Abstract

**Background:**

Peer support involves people (mentors) using their own experiences to assist others (mentees). The impetus to include peer support in eating disorder recovery is high, however research on implementation of peer roles in eating disorder management is limited. A previous pilot study found positive but preliminary results for a Peer Mentor Program (PMP) for eating disorders. The PMP has since developed over time, including broadening its eligibility criteria and shifting to predominantly online delivery during COVID-19. This study aimed to evaluate the updated version of the PMP, on a larger and more diverse group of mentees.

**Methods:**

Previously collected PMP service data from July 2020 to April 2022 (during COVID-19 lockdowns) was evaluated for fifty-one mentees using mixed methods. Data from program start (baseline), mid-point (3-months) and end (6-months) for measures of eating disorder symptoms as measured by the Eating Disorder Examination Questionnaire (EDE-Q) and psychological wellbeing as measured by the Depression, Anxiety and Stress Scale (DASS) was evaluated. Frequency of eating disorder-related hospital admissions during PMP participation versus the 6 months prior, direct program costs and qualitative mentee feedback were also analysed. One way ANOVA’s with post hoc tests were used to evaluate symptom change and thematic analysis was conducted on qualitative data.

**Results:**

Program attendance averaged 12.12 (*SD* ± 1.57) of a possible 13 sessions. Statistically significant and clinically meaningful improvements were demonstrated across all subscales of the eating disorder and psychological wellbeing symptom measures. EDE-Q Global score and DASS scores decreased significantly by program end. Fewer eating disorder-related hospital admissions were reported during PMP than the 6-months prior. Qualitative findings were positive and themed around the unique benefits of lived experience connection, a new kind of space for recovery, hope and motivation for change. Challenges with the time limited nature of the mentee-mentor relationship were expressed.

**Conclusions:**

The important benefits of a PMP for individuals with eating disorders are further supported. There is a pressing need for high quality, co-produced research, utilising a mixture of designs and fidelity to core peer work principles, to inform further implementation of peer work into eating disorder policy and practice.

**Supplementary Information:**

The online version contains supplementary material available at 10.1186/s40337-024-01051-7.

## Abstract (plain language summary)

Peer mentoring involves people using their personal lived experiences to support others. A pilot Peer Mentoring Program for eating disorders (PMP) offered through Eating Disorders Victoria (EDV) was evaluated previously, showing positive findings. Subsequent demand for the program was high. As a result, the PMP has been running and evolving over time. Key changes included a broadening of program eligibility to all individuals with an eating disorder (versus only people recently discharged from hospital), larger participant numbers and a shift to largely online delivery due to COVID-19. This study evaluated this current version of the PMP. Five rounds of anonymised PMP data, from July 2020 to April 2022, were evaluated with mentee prior consent. Individuals in recovery from an eating disorder (mentees), had been paired with individuals recovered from an eating disorder for 2-years minimum (mentors). PMP rounds were 6-months, with fortnightly meetings. Mentees overall showed improvements in eating disorder symptoms and psychological wellbeing. Fewer mentee eating disorder-related hospital admissions during PMP participation were reported, compared to the 6-months prior. Feedback from mentees identified many positive benefits and some challenges. Overall, the results provide further support for the use of peer mentoring in eating disorder recovery.

**Supplementary Information:**

The online version contains supplementary material available at 10.1186/s40337-024-01051-7.

## Background

Eating Disorders are common, costly and disabling [1–3]. All eating disorder diagnoses are associated with elevated psychosocial and medical comorbidity, with anorexia nervosa having the highest mortality rate of all mental illnesses [4, 5]. Eating disorders occur irrespective of age, gender, cultural background, sexual orientation and body size, with important groups being at elevated risk including Aboriginal and Torres Strait Islander Peoples (First Australians) [6], LGBTIQA + individuals [7], neurodiverse individuals [8] and people experiencing certain physical conditions such as diabetes [9]. As many as 80% of people with an eating disorder do not seek eating disorder-specific interventions, and for those who do, treatment can be substantially delayed, poorly co-ordinated, or inadequately aligned to individual needs [10]. Treatment relapse rates are high, averaging between 30% and 50% depending on diagnosis when followed for 10 years [11]. Taken together, there is a clear and urgent need for diversification, innovation and improved accessibility of person-focussed eating disorder treatments.

Peer support, the sharing of related lived experience in order to connect, support and learn with and from others, offers significant benefits within mental health [12]. Based on “a system of giving and receiving help founded on key principles of respect, shared responsibility and mutual agreement of what is helpful” [13], (p 135), peer support is relational in nature. Whilst underpinned by complex mechanisms, peer support operates through mutual challenge and personal growth, it mirrors naturally occurring peer support relationships and aims to build hope, empowerment, social inclusion and self-management [12, 14]. Positive outcomes from peer support in mental health have been demonstrated, including in early psychosis intervention [15], for reducing psychiatric admissions [16] and assisting people with complex needs to engage in services [17]. With the current policy landscape advocating for lived experience in the co-production and co-delivery of eating disorder services, there is strong impetus to build an evidence base to support safe and effective implementation [18]. Eating Disorders Peer Workforce Guidelines have recently been released in Australia, which is a promising advancement [19]. Research in the context of peer support programs in eating disorders is, however, currently limited and outcomes have been mixed [18, 20].

In terms of the extant literature, Ramjan et al. (2017) ran a proof-of-concept study on a mentoring support program for people with any eating disorder *(n* = *10 mentees)* [21]*.* They reported statistically significant improvements in hope for recovery in the domains of social relationships, romantic relationships, family life, work and overall scores. Ramjan et al. (2018) followed by using a mixed methods, participatory action research design to study a 13-week face-to-face community-based peer mentoring program for anorexia nervosa (*n* = *6 mentees*) [22]. They reported on the importance of connecting people in recovery with “someone who understands” to facilitate hope, grow relationships and build quality of life. A randomised controlled trial of a face-to-face hospital-based peer mentoring program for a range of eating disorder diagnoses (anorexia nervosa, bulimia nervosa and binge-eating disorder) (*n* = *60*), showed reductions in body dissatisfaction, symptoms of depression and anxiety, and frequency of binge eating and restriction [23]. Peer-mentorship did not impact re-entry into higher level of care or body mass index. Finally, a 6-month community-based Peer Mentoring Program (PMP) in eating disorders was piloted by a research team in collaboration with Eating Disorders Victoria (EDV), the largest community-based organisation providing eating disorder specific services in Victoria, Australia [24–26]. A further evaluation of EDVs PMP is the focus of this current study.

The PMP was designed to address a service gap for people requiring additional support after an eating disorder-related hospital admission [24]. The PMP uses a peer support model, harnessing the experience of people who had recovered from an eating disorder (mentors) to support people in recovery (mentees) as an adjunct to their eating disorder treatment. The initial pilot PMP evaluation engaged mentees (*n* = *22*) who had recently been discharged from a high acuity eating disorder-related admission and showed promising preliminary results, indicating “moderate feasibility” [25]. By program end, mentees demonstrated on average modest improvements in body mass index, quality of life, eating disorder symptomatology, depression, anxiety, stress and perceived disability. Important qualitative themes arose from program participation including hope for recovery, greater personal agency and inspiration from first-hand interaction with another person who had recovered from an eating disorder [26]. With additional funding, EDV went on to run their highly sought after PMP in the community for over four years with the current evaluation arm possible by translational research funding (MRFF Million Minds - MAINSTREAM).

Since the initial pilot, EDV’s PMP has evolved. Over 150 mentees have now completed the program. PMP improvements include enhanced mentor training and supervision, refined program systems and procedures, and greater staff expertise using intentional peer support in eating disorder recovery. PMP eligibility broadened to include anyone in the community 18 years of age or over seeking support for eating disorder recovery, rather than only those with a recent hospital stay only. Furthermore, the PMP shifted to predominantly online delivery (i.e., fully online or online with minimal in-person meetings outdoors) in response to COVID-19 lockdowns imposed by the Victorian State Government during the 2020–2021 pandemic years, which also increased geographical reach.

The primary aim of this study was to evaluate the updated PMP for eating disorders. We hypothesised that this program would be associated with reductions in mentee eating disorder symptomatology and lowered symptoms of depression, anxiety and stress, when delivered online as part of community-based eating disorder management. We further aimed to compare the frequency of mentee eating disorder-related hospital admissions in the 6-months prior to the PMP, versus during the PMP period, and to report direct program costs. A secondary study aim was to continue to build our understanding of mentee experiences of the PMP from their feedback, using qualitative thematic analysis.

## Method

### Study design, time frame and setting

This study employed a single-site, retrospective, pre-post, uncontrolled, service evaluation design using mixed methods [27]. Retrospective ethical approval was granted by the Bellberry Human Ethics Research Committee (HREC 2022_04_374). Service delivery and evaluation data collected across 5 rounds of the PMP (from July 2020 to April 2022) were pooled for analysis. The PMP ran from the EDV offices in Melbourne, Australia and sessions were conducted across various formats and locations in keeping with COVID-19 lockdown rules, including online and at times suitable outdoors locations (e.g., parks). Meetings were not permitted in mentor or mentee homes.

### Participants and procedures

Participants were mentees who undertook EDV’s PMP during the study timeframe. PMP recruitment was from a broad range of sources where the program was promoted, including hospital and medical/mental health practitioners, as well as EDV’s website, social media and newsletter. PMP staff, that is EDV staff who oversaw administration of the program, assessed program eligibility based primarily on verbal self-reported information from potential participants. PMP staff were all trained in mental health and eating disorders, with some having related lived experience. Mentors are not the focus of this current evaluation, however they were individuals who had recovered from an eating disorder for a minimum of two years duration and acted as peer mentors in the program. All mentors had undergone three days of intensive training and an induction run by EDV, prior to being matched with a mentee. EDV provided all mentors with regular de-briefing and bi-monthly group supervision throughout each of the rounds of the PMP in this study.

Inclusion criteria for PMP participation (and the study) were: being 18 years or older; a self-reported current eating disorder diagnosis as indicated by their health care team (including anorexia nervosa, bulimia nervosa, binge eating disorder, atypical anorexia nervosa or avoidant restrictive food intake disorder); and actively engaged in community-based eating disorder management including a treating general practitioner, a mental health practitioner and a personal support person for the duration of the PMP. A personal support person is someone who a mentee nominates that is aware of their diagnoses and who they would be comfortable with EDV contacting if there was a concern about their health or wellbeing. Once nominated, it was assumed that this person would be available for the mentee for the duration of the PMP*.* A personal support person can be a family member, loved one or friend. Exclusion criteria (for PMP enrolment and thus for this evaluation) were past participation in the program, serious acute risk of harm to oneself or another (e.g., individuals at high risk of suicide) and/or being a current inpatient at the time of program start. Mentees were not excluded from the PMP if they underwent a hospital admission during the program, mentees were offered the option to continue, or pause, mentor sessions whilst an inpatient.

In total, 117 people were assessed for eligibility for EDV’s PMP running between July 1, 2020 to April 31, 2022 (i.e., the five rounds of PMP being retrospectively analysed in this study) and 87 were deemed eligible and enrolled. Six people did not proceed, with baseline program data subsequently collected for 81 people (i.e., mentees). Seventeen mentees (21%) withdrew for a variety of personal, clinical and program reasons (e.g., insufficient time, alternative treatments or deeming program ‘fit’ was not right). One significant outlier was removed for analysis due to invalid questionnaire scores.

**Fig. 1:**
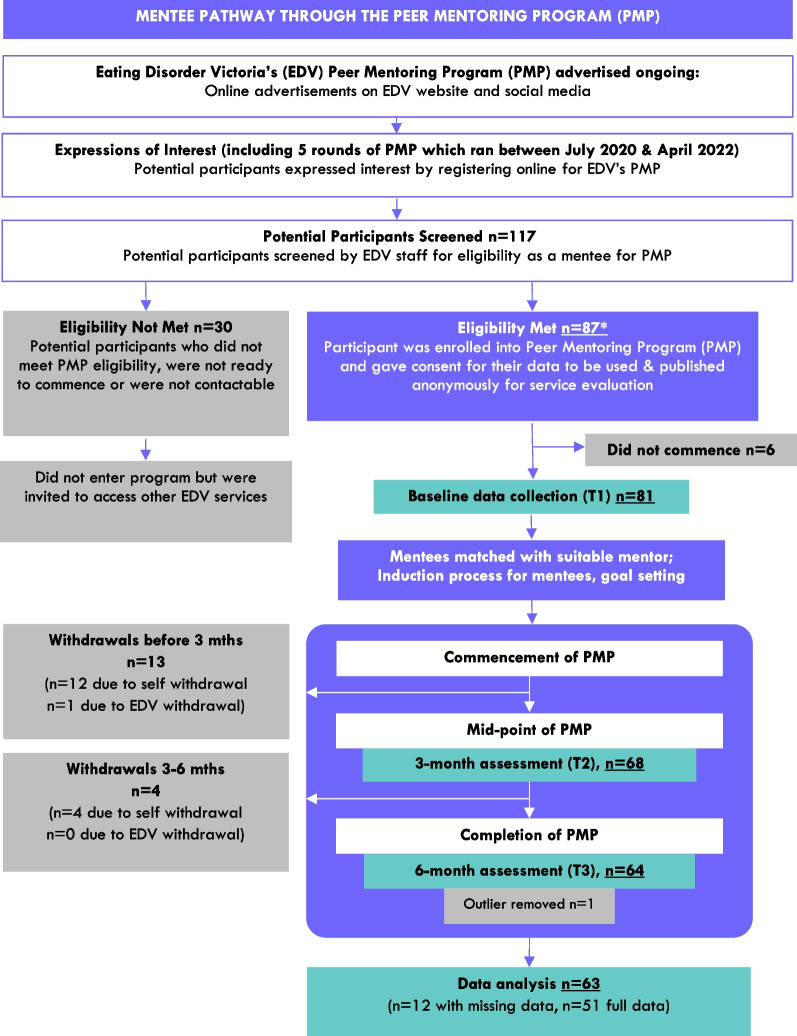
Flowchart of mentees through the Peer Mentoring Program (PMP) across the study timeframe.*NB - 87 participants (mentees) in total were enrolled into the Peer Mentoring Program (PMP) between July 1 2020 and April 31, 2022. This flow chart shows the process followed for each of the 5 rounds over this time period, with total participants numbers (n) summed

Of the remaining 63 mentees, complete data were available for 51 (81%) as primary outcome data were missing at either one or two time points for 12 (19%). A comparison between baseline characteristics of mentees with complete data (*n* = 51) versus missing data (*n* = 12) identified a possible difference in eating disorder diagnostic profile, suggesting data may not be missing at random. For example, in those with missing data there was a relatively higher rate of bulimia nervosa (25% compared to 7.8%) and eating disorder duration was on average five years longer. Data imputation was not deemed appropriate and therefore the 51 cases with full data on all primary outcome measures were included for analysis (Fig. 1).

### Program details

EDV’s PMP involved fortnightly individual mentoring sessions over 6 months. Mentees attended an average of 12.1 (*SD* ± 1.6) PMP sessions out of a possible 13 sessions (range 6 to 13) indicating overall high attendance (87% attended over 75% of PMP sessions). A detailed program description is provided in Additional File 1. Robust consent processes are in place at EDV for collection, analysis and reporting of service user data. Prior written approval was given by all mentees whose data was subsequently de-identified and pooled in the current analysis. Service data was not collected anonymously but was de-identified by EDV staff prior to analysis commencing. No mentees opted out, or requested withdrawal, of their de-identified data for service evaluation and publication. Program evaluation data was collected using FormAssembly, an online secure data storage system. All data were stored in EDV’s online, secure data storage system.

### Program evaluation approach

#### Demographic and clinical information

Demographic and clinical information, including age, identified gender, work status, education status and eating disorder diagnosis and duration, were collected via online self-report prior to PMP commencement.

#### Quantitative evaluation: eating disorder symptomatology and psychological wellbeing questionnaires

Mentees completed two questionnaires to evaluate change in eating disorder symptomatology and psychological wellbeing across PMP participation. These were utilised in two ways: (1) to monitor mentee wellbeing to guide if program modifications were needed, and (2) for program evaluation. Questionnaires were administered via automated email at program start, mid and end (i.e., baseline, 3-months and 6-months). The questionnaires were the Eating Disorder Examination Questionnaire (EDE-Q) [28] and the Depression, Anxiety and Stress Scale (DASS-21) [29]. Both have acceptable to good psychometric properties and are described elsewhere in detail [30–33]. Higher scores indicate higher symptomatology.

#### Eating disorder-related hospital admission data before vs during PMP

Self-reported, eating disorder-related inpatient hospital admission rates (medical or psychiatric admissions specific to the eating disorder) for the 6-months prior to PMP participation were collected during mentee online registration. Inpatient eating disorder-related admission rates during the 6-months of program participation were recorded by EDV program staff.

#### Peer mentoring program (PMP) direct costs

Direct costs of the PMP for the study period were derived from EDV operating budgets from July 2020 to April 2022. Expense categories included: general administration, infrastructure and information technology and program-related costs.

#### Qualitative evaluation: mentee feedback

Mentees provided online written feedback at mid-program and program end. The questions asked were what mentees (1) enjoyed most and found the least challenging, and (2) enjoyed the least and found most challenging, about PMP participation. Mentee feedback was used to optimise program responsiveness to their expressed needs and for service evaluation and quality improvement.

### Statistical analysis

IBM SPSS Statistics (version 28.0) was used for quantitative analyses. Descriptive statistics were calculated. One-way repeated measures analyses of variance (ANOVA) tests evaluated change on EDE-Q and DASS scores across the 3 time points. Statistical assumptions were met for the one-way repeated measures ANOVA tests, except for the sphericity assumption for the EDE-Q Shape Concern and EDE-Q Weight Concern subscales, where a Greenhouse–Geisser correction was applied [34]. Bonferonni adjusted post-hoc tests evaluated specific time frames in which scores EDE-Q or DASS scores changed (i.e., baseline and 3-months or 3-months and 6-months). A *t*-test was used to compare differences in frequency of mentee eating disorder-related hospital admissions in the 6 months prior compared to during the PMP period. All tests were conducted using an alpha of 0.05.

### Qualitative analysis

Qualitative analysis followed a six-stage approach of thematic analysis [35] to identify key themes in mentee feedback data. This analysis involves six phases: 1. familiarisation with the data, 2. generating initial codes, 3. searching for themes, 4. reviewing themes, 5. defining and naming themes and 6. producing the report. Qualitative analysis was overseen by the first author (AR) in collaboration with co-authors (RD & AS). Initial coding was undertaken manually by the first author on the entire data set, drawing on inductive approaches. This stage involved deep engagement with the data, to identify repeated patterns in mentee feedback. Codes were constructed around the two key feedback questions posed (i.e., 1. *What were the most enjoyed/least challenging aspects of the PMP?* and 2. What were the *least enjoyed/most challenging aspects of the PMP?)* and codes were generated in response patterns which were conceptualised from the data, strongly grounded in mentees’ accounts. Codes were grouped into themes and subthemes based on all feedback provided by the 51 mentees. The coding framework was refined over iterations in consultation between AR, RD & AS, as coded material was re-considered, similar codes merged and obsolete codes were deleted. Themes were therefore reviewed and amended to ensure they formed a consistent and authentic representation of both coded extracts and the entire feedback data set. This research was conducted primarily out of Eating Disorders Victoria (EDV), in conjunction with the MAINSTEAM collaboration and the research team, whom seek to improve care offered to people in recovery from an eating disorder. EDV’s vision is of “a future where individuals and communities thrive through empowered and safe relationships to food, eating, body and movement” and EDV’s services are strongly informed by people who have lived experienced of eating disorders and their loved ones.

First author AR, is a researcher, academic and mental health clinician working in the field, she did not work or have direct involvement with running the PMP at any stage of this evaluation (i.e., program delivery, data collection and analysis). AR contributed qualitative and quantitative research methods and service evaluation experience to the current study. As senior EDV staff members, authors RD and AS bought backgrounds in mental health work, including experience in peer work, program design & implementation and in overseeing the coordination of the PMP. They were both senior staff members in the PMP at the time of data collection, analysis and manuscript editing. Overall, the multidisciplinary research team broadly bring a range of different perspectives to this evaluation, including clinical, research, service provision (public community, hospital & private sector) and translation backgrounds. This is the first formal evaluation study of the PMP that AR, RD and AS have been involved in. Several of the authors were involved in the conduct and publication of the original PMP pilot studies (AP, DC, RN, LB, ZJ) [25, 26].

## Results

### Demographic and clinical information

Mentee baseline characteristics are provided in Table 1. Mentees averaged 27 years of age (*SD* ± 8.7) and predominantly identified as women (94.1%). The primary self-reported eating disorder diagnosis was anorexia nervosa (AN; 76.5%), followed by binge eating disorder (BED; 11.8%) and bulimia nervosa (BN; 7.8%) being the next most frequent eating disorder diagnoses.

**Table 1:** Baseline characteristics of mentees upon registration into the Peer Mentoring Program (PMP) (n = 51)

Baseline characteristic*	Mean, ± SD or Number, %
Age, years *(Mean,* ± *SD)*	
	27.2 (8.7)
Gender *(Number, %)*	
Woman	48 (94.1)
Employment *(Number, %)*	
Full-time	12 (23.5)
Part-time or casual	16 (31.4)
Student	13 (25.5)
Not employed	10 (19.6)
Highest education *(Number, %)*	
Bachelor degree or Postgraduate	26 (51.0)
Certificate III/IV or Diploma	13 (25.5)
Year 11/12	12 (23.5)
Eating disorder diagnosis - primary* (Number, %)*	
Anorexia Nervosa (AN)	39 (76.5)
^#^Binge Eating Disorder (BED), Bulimia Nervosa (BN),	12 (23.5)
Atypical Anorexia Nervosa (OSFED: AAN) or Avoidant Resistant Food Intake Disorder (ARFID)	
Eating disorder duration, years *(Mean* ± *SD)*	
	9.2 (9.5)

Data analysis was conducted on mentees (n = 51) for whom there was no missing data on primary outcomes and who did not withdraw. Those who did not complete all 13 sessions are still included in the analysis. All data including eating disorder diagnosis was self-reported by mentees in the Peer Mentoring Program (PMP) online registration forms, which were complete just prior to program commencement

*Cell sizes of < 10 are not reported to protect confidentiality of participants

^#^For each of these eating disorder diagnoses n was ≤ 4 participants therefore diagnostic groups were collated

### Quantitative evaluation: changes in eating disorder symptomatology and psychological wellbeing

**Fig. 2:**
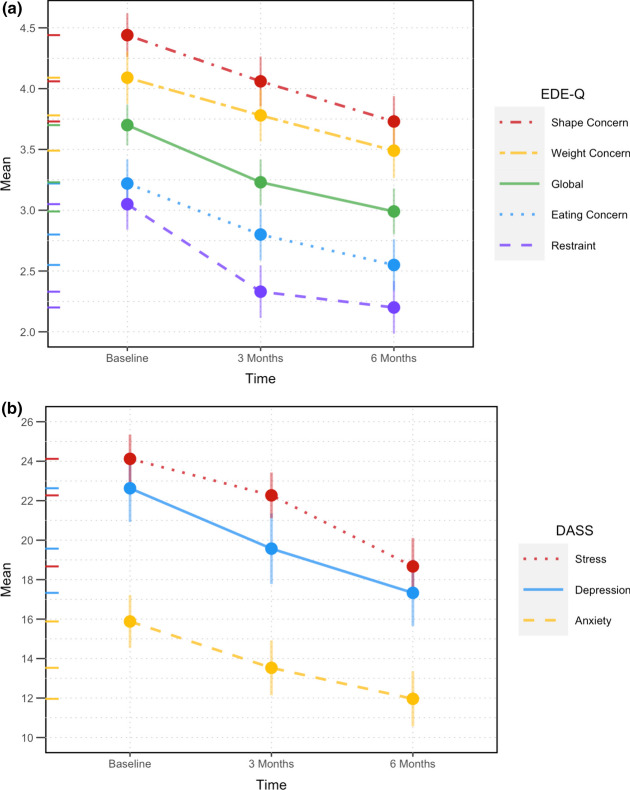
Change in eating disorder (EDE-Q) and wellbeing (DASS) symptoms across participation in the Peer Mentoring Program (PMP) (n = 51). **a** Changes in Eating Disorder Examination Questionnaire (EDE-Q) subscales and global score across PMP participation. **b** Changes in Depression, Anxiety and Stress (DASS) subscales across PMP participation

Overall, there was a statistically significant decrease in scores (improvement) across the 6-months of PMP for all primary outcome measures - the Global EDE-Q score, as well as its constituent subscales Restraint, Eating Concern, Weight Concern & Shape Concern, and the Depression, Anxiety and Stress subscales of the DASS. Effects sizes found were mostly in the statistically large range according to Cohen (1988). Refer to Table 2. The EDE-Q Global score and EDE-Q Restraint and Eating Concern sub-scale scores had decreased significantly by 3-months of the PMP and between baseline and 6-months. The EDE-Q Shape Concern sub-scale reduced by 3-months, between 3-months and 6-months and between baseline and 6-months. All three DASS subscales reduced significantly but only between baseline and 6-months (i.e., program end), except DASS-Stress which also decreased significantly between 3- and 6-months. DASS-Anxiety and DASS-Depression each reduced from the ‘severe’ to the ‘moderate’ category and stress scores changed from ‘moderate’ to ‘mild’ [36]. Refer to Table 3 and Fig. 2a & 2b.

**Table 2:** Peer Mentoring Program (PMP) measures at baseline, 3-months and 6-months (n = 51)

Outcome Measure	Baseline *(M/SD)*	3-months *(M/SD)*	6-months *(M/SD)*	*p* value	Effect size^	*Mean* diff^#^	95% CI of Mean diff
EDE-Q Restraint	3.05 (1.50)	2.33 (1.54)	2.20 (1.54)	< .001^†^	.201	0.85	.35 to 1.36
EDE-Q Eating Concern	3.22 (1.42)	2.80 (1.51)	2.55 (1.51)	< .001^†^	.146	0.67	.25 to 1.10
EDE-Q Shape Concern	4.44 (1.28)	4.06 (1.46)	3.73 (1.49)	< .001^†+^	.183	0.71	.28 to 1.15
EDE-Q Weight Concern	4.09 (1.54)	3.78 (1.53)	3.49 (1.61)	.003^†+^	.117	0.60	.12 to 1.01
EDE-Q Global Score	3.70 (1.19)	3.23 (1.34)	2.99 (1.35)	< .001^†^	.223	0.71	.34 to 1.08
DASS Depression	22.63 (12.21)	19.57 (12.81)	17.33 (11.99)	.003^†^	.112	5.29	1.45 to 9.13
DASS Anxiety	15.88 (9.53)	13.53 (9.91)	11.96 (9.98)	.004^†^	.103	3.92	.69 to 7.15
DASS Stress	24.12 (8.75)	22.27 (8.26)	18.67 (10.27)	< .001^†^	.196	5.45	2.38 to 8.53

Data analysis was conducted on mentees (n = 51) for whom there was no missing data on primary outcomes and who did not withdraw. Those who did not complete all 13 sessions are still included in the analysis; *p* values are from repeated measures ANOVA

^†^denotes statistical significance at .05 level

^+^denotes Greenhouse–Geisser correction

^denotes Partial Eta Squared with .01 = small, .06 = moderate and .14 large effect sizes (Cohen, 1988); Mean diff = mean difference

^#^denotes mean difference in the respective score from baseline to 6-months

**Table 3:** Post-hoc comparisons between Peer Mentoring Program (PMP) assessment measures at baseline, 3-months and 6-months (n = 51)

	Baseline & 3-months		3-months & 6-months		Baseline & 6-months	
Outcome Measure	*Mean* diff^#^	*p* value^b^	*Mean* diff^#^	*p* value^b^	*Mean* diff^#^	*p* value^b^
EDE-Q Restraint	0.72	< .001^†^	0.14	1.000^NS^	0.85	< .001^†^
EDE-Q Eating Concern	0.43	.030^†^	0.25	.42^NS^	0.68	< .001^†^
EDE-Q Shape Concern	0.38	.035^†^	0.33	.034^†^	0.71	< .001^†^
EDE-Q Weight Concern	0.30	.106^NS^	0.29	.181^NS^	0.60	.010^†^
EDE-Q Global Score	0.47	< .001^†^	0.24	.221^NS^	0.71	< .001^†^
DASS Depression	3.06	.064^NS^	2.24	.527^NS^	5.29	.004^†^
DASS Anxiety	2.35	.056^NS^	1.57	.582^NS^	3.92	.012^†^
DASS Stress	1.84	.220^NS^	3.61	.006^†^	5.45	< .001^†^

*p* value^b^ denotes p values with Bonferroni adjustment for multiple comparisons

† denotes statistical significance at .05 level

^NS^denotes non-significance

### Eating disorder-related hospital admissions before v’s during PMP

Thirty-one eating disorder-related hospital admissions were self-reported in the 6 months prior to participation in the PMP across 25 individuals, with 21 mentees reporting one admission (41.2% of mentees) and the remainder reporting two or more admissions (7.8% of mentees). Fewer eating disorder-related hospital admissions occurred during PMP, with 11 admissions in total (i.e., 20 less admissions). Of those 11 admissions, nine mentees reported one admission (17.6% of mentees), and the remainder reported two or more admissions (2.0% of mentees). It was the first eating disorder-related admission for a small number of mentees and a re-admission for eight.

Regarding hospital admissions before versus during the PMP, twenty-four mentees (47.1%) did not report a hospital admission in either the 6-months before or during PMP participation and eight mentees (15.7%) reported a hospital admission in both the 6-months before and during PMP participation (i.e., no change). Two mentees (3.9%) had a hospital admission during PMP participation yet not before PMP (i.e., increase in hospital admissions), however, seventeen mentees (33.3%) reported a hospital admission in the 6-months before but not during PMP (i.e., decrease in hospital admissions). Overall, there were 31 admissions between 51 participants in the 6-months prior to PMP, (representing a 0.61 likelihood of hospital admission), however only 11 admissions during PMP (0.22 likelihood of hospital admission).

### Peer mentoring program (PMP) direct costs

Direct costs of the PMP totalled $840,090 AUD across the study period. This equated to an average of $168,018 per round and $10,372 per mentee. Average cost per mentee was calculated for the 81 mentees who commenced the PMP in the study period, to also account for costs incurred by the service for those participants who withdrew from the program early (n = 17). This figure for direct costs included general administration costs, such as office costs, infrastructure and IT including rent and internet, program specific costs for mentees such as the costs of mentee-mentor activities undertaken and program specific costs for mentors including remuneration and supervision, staff costs and program administration. While the majority of the PMP was delivered online for the rounds of the PMP reported herein, office space, IT and internet costs for staff were still incurred. Of note is that the biggest expense of running the PMP, was for PMP staff (program & administrative), mentee and supervisor remuneration costs. A breakdown of costs for the Peer Mentoring Program according to key categories is provided in Table 4. Additional costs (such as hospital stays) were beyond the scope of this study.

**Table 4:** A breakdown of costings for the Peer Mentoring Program (PMP)

Resource Item	Total cost (5 rounds of PMP)*	Average cost per study PMP round	Average cost per mentee (n = 81)^#^
General administration, e.g., office costs	$44,183	$8,837	$546
Infrastructure & IT, e.g., rent, internet	$39,042	$7808	$482
Program costs - mentees e.g., activity costs	$25,206	$5041	$311
Program costs - mentors, staff, supervision, administration	$727,337	$145,647	$8980
TOTAL COSTS (AUD)	$840,090	$168,018	$10,372

*Taken across the study time period of July 1, 2020 to April 31

^**#**^data is presented from n = 81 as this was the total number of mentees who commenced the PMP across the 5 study rounds, thereby includes program costs for people who completed the program (n = 64) and accounts for costs incurred from program withdrawals (n = 17)

### Qualitative evaluation: mentee feedback

Below are findings and sample quotes from a cross-section of mentee responses (using initial-based pseudonyms), derived from the thematic analysis of PMP mentee written feedback. A summary of the feedback questions with a collated list of themes can be found in Table 5.

**Table 5:** Peer Mentoring Program (PMP) a summary of key qualitative feedback questions and related themes

**Feedback Question 1 - Most enjoyed/least challenging aspects of the PMP**
Theme 1.1: Power of the lived experience connection
Theme 1.2: A new kind of recovery space
Theme 1.3: Strengthening the foundations for recovery
Theme 1.4: Reaping the rewards of “going there”
**Feedback Question 2 - Least enjoyed / most challenging aspects of the PMP**
Theme 2.1: The double-edged sword of “going there”
Theme 2.2: Vulnerability and trust
Theme 2.3: Navigating closeness with a trusted stranger
Theme 2.4: Logistics matter

#### Feedback Question 1 - Most enjoyed/least challenging aspects of the PMP

##### Theme 1.1: Power of the lived experience connection

Mentees reflected personal benefit from connecting first-hand with someone who had recovered from an eating disorder and was open to sharing their experiences.

“Hearing my mentor’s story helped me see that there is a life outside of an eating disorder and that full recovery is possible”(FT)

Mentees further described positive impact from feeling heard, understood, validated and guided in a nuanced way.

“Spending time talking to someone else who understands completely what I'm going through and is empathetic, sharing experiences, doing things that I would have been scared to try alone but have been really beneficial for me (e.g. yoga), having a supportive person who cares about me.”(DE).

Some mentees identified value in someone understanding and caring about their daily struggles with an eating disorder, and who in turn was there to support them trying new experiences.

“I enjoyed being able to talk to my peer mentor about struggles and difficulties I was experiencing and gaining support from a person with lived experience. I also enjoyed being able to try new things, challenge myself in a safe and supportive environment.”(NJ).

##### Theme 1.2: A new kind of recovery space

Mentees identified that the PMP offered a different kind of therapeutic space to explore recovery. This included authenticity from mentors and a safe space to explore any topic without fear of judgement or shame.

“Having someone with lived experience to talk to about my fears, concerns and eating disorder behaviours without fear of judgement. I felt as though I could ask anything and together we would work towards a solution.”(NY).

Mentees welcomed the opportunity to discuss experiences with someone who had firsthand experience of the challenges of living with an eating disorder.

“My mentor’s incredible understanding of living with an eating disorder. Laughing! The feeling of looking forward to our meetings and comfort, when I have felt hopeless, that I was not alone.”(TI).

More equal power dynamics, connecting with someone to whom mentees could relate (e.g., closer in age and interests), were reflected as helpful and additional to past treatment set ups.

“Having the opportunity to connect with a peer of similar age and positionality who also has the capacity and understanding to support me in the contexts of dining out and more day-to-day eating disorder recovery factors than I have had the opportunity to delve into in other treatment contexts.”(MH).

##### Theme 1.3: Strengthening the foundations for recovery

Themes of inspiration and hope for recovery were relayed by mentees.

“Getting out and about which I never really do, and having someone to chat to that has somewhat recovered from an eating disorder gives hope that it might be possible for me to improve my quality of life in that area too.”(TG).

Greater connection to others, belonging to a group, increased positive social engagement and reduced isolation, were factors identified in enabling positive change.

“It's also been really nice to feel part of something, and part of some kind of community through the program.”(OQ)

Key messages towards re-discovering sense of self-identity, self-worth and connecting with life motivations for recovery were relayed, for example, *“finding myself again(WO)“*, *“feeling like a normal person”(IY)* and *“seeing there is a life outside of an eating disorder”(FT).*

##### Theme 1.4: Reaping the rewards of “going there”

Mentees commented on the power of mentor support to help drive positive change and the subsequent rewards, as akin to being pushed out of the comfort zone but in a helpful way.

“Getting to experience some real-world activities outside of my comfort zone with lots of support and having someone to share anything with and get advice without feeling like I am being forced to do anything.”(FF).

“Having moral support and a friend for challenging situations outside of my comfort zone, which I never would have otherwise tried on my own.”(TU)

Help with goal setting and problem solving the ‘how to’ practical aspects of eating disorder recovery were valued by mentees (e.g., planning for social events, cooking together and help undertaking food challenges).

“Making challenges to complete between sessions became a really useful tool and accelerated my progress in recovery.”(NY)

Additionally, positive impacts of building motivation experientially, with persistence and help, was relayed as beneficial.

“The feeling of quiet accomplishment after trying something that previously scared me, like eating out socially in such a "normal" way.”(TU)

#### Feedback Question 2–Least enjoyed/most challenging aspects of the PMP

##### Theme 2.1: The double-edged sword of ‘going there’

Whilst mentees clearly reported benefits from increased engagement with the tasks of recovery, this was also reflected as one of the greatest challenges of the PMP.

“Sort of the reverse of the above, pushing myself to open up has been incredibly challenging however, it's also been incredibly rewarding thus a huge positive for me.”(FJ)

Translating learnings into life, choosing recovery and doing the work were commonly relayed concepts that were expressed as very difficult, however potentially reflect a strength of the program in supporting individuals enact change.

“The parts that I enjoyed the least were the struggles of choosing recovery and doing activities that challenged my eating disorder, however it always ended up being beneficial.”(KI).

##### Theme 2.2: Trust and vulnerability

A consistent theme was around the challenges of allowing oneself to trust the program as a safe space to be open and vulnerable, despite a time limited arrangement.

“At first it was hard to be open and honest as we didn’t know each other”(KT)

“It was challenging to open up because I hate being vulnerable.”(CU)”

Opening up to big emotions involving sharing feelings, values, fears and wants, came with trepidation, but also built hope for some mentees. Leaning into confronting experiences such as “*realising how far I had to go”(CO),* managing *“perfectionis*tic personal challenges*”(UQ)* and “*drawing comparisons to others in the program”(MQ)* were reflected as valuable but challenging aspects of participation.

##### Theme 2.3: Navigating closeness with a trusted stranger

The unique mentee-mentor relationship was portrayed by mentees as a beneficial and fundamental program feature. This relationship, however, came with challenges around learning to navigate a new kind of relationship - one which was neither completely personal, nor clinical. Seeking to understand the boundaries of the mentee-mentor relationship (e.g., what can I ask, what can I say) and learning to maximise the available time, were two of the issues raised.

“I've found it a bit challenging to ask more personal questions, such as how to navigate an eating disorder in a relationship or how to talk about it with my partner, because it's a personal question and because I don't want to overstep my mentor's boundaries.”(TU).

“Sometimes I feel I am imposing when I call or text at challenging times.”(WO)

The inevitable parting of ways with a mentor instilled sadness and a sense of vulnerability in some mentees, reflected in their feedback.

“The wave of sadness, grief and loss that has come with the end of the program and trying to navigate my way through these emotions.”(MQ)

##### Theme 2.4: Logistics matter

The shift to predominantly online delivery of the PMP due to COVID-19 raised a unique situation in the program’s history. No single preferred delivery mode was identified, but a greater variety of delivery options provided versatility to individual mentee needs and preferences. Some reported the online only mode reduced capacity for connection with their mentors, while others reported that flexible delivery enabled them to participate even if geographically constrained (e.g., if in hospital or outside of the metropolitan area).

“I found it challenging being confined to zoom and phone calls only. I would have loved to of met my mentor face to face but that didn't end up happening due to a variety of reasons.”(FT) versus “Flexibility of sessions and allowing it to fit into my lifestyle with work.”(FJ).

## Discussion

The overarching purpose of this study was to further build understanding of the effectiveness and acceptability of EDV’s PMP, a now well-established community program. We utilised a mixed method design, that is, an approach which utilised the available program quantitative and qualitative data [27], to gain a fuller understanding of the ways in which mentees experienced the PMP. An additional aim was to interpret the impact of a shift of PMP to online delivery, in context of the significant challenges of extended COVID-19 lockdowns on the physical and mental wellbeing of local communities [37]. The diversity of ages and eating disorder diagnosis duration in the mentees included is wider, suggesting good program adaptability and inclusivity. Acceptability of the program was high, as reflected by consistent mentee attendance, expressed program enjoyment and the range of benefits experienced.

The first study aim was to evaluate whether program participation was associated with changes in eating disorder symptomatology and psychological wellbeing. The EDE-Q Global score and the DASS subscales reduced around 20% on average, representing both statistically significant and clinically meaningful improvements [36, 38]. These outcomes are particularly important given the data was collected across the very challenging time of COVID-19 lockdowns when the risk of eating disorder, depression and anxiety symptoms worsening was significantly elevated [39].

A further feature of this study was the access to three data points (i.e., baseline, 3-months and 6-months), providing information on the timing of symptom changes. Regarding the EDE-Q, the Restraint subscale showed the largest change particularly in the first 3-months, with the Eating Concern subscale changing in a similar pattern but to a lesser magnitude. It may be that eating concerns potentially reduced, as behavioural changes around restraint provided corrective experiences via challenging eating anxiety. The Shape Concern subscale appeared to improve a modest amount by 3-months and 6-months, but this equated to a statistically significant change by program end. The Weight Concern subscale demonstrated the least change and appeared to take longer to show improvements, albeit reaching significance by program end. This is not unexpected as body image often takes time to change particularly in clinical level eating disorders [40].

Statistically significant reductions in symptoms of depression, anxiety and stress, occurred in a pattern of reduction across the entire program. The exception was the Stress subscale, which also indicated a pattern of change between program mid-point and end. We interpreted this change as a clinically meaningful, as according to symptom severity scores for the DASS [36], depression and anxiety scores in the current study reduced from the ‘severe’ to the ‘moderate’ category and stress scores reduced from the ‘moderate’ to ‘mild’ category. Taken together, we believe these reductions in symptomatology are also likely to represent meaningful positive improvements to mentees lives and capacity for recovery, given there is a well-documented link between increased mental health concerns and eating disorder symptomatology, and consequently improved mental health with eating disorder recovery [41].

Of note is that the reported changes in eating disorder and psychological wellbeing symptom scores represent average improvements. There were a range of responses to the program; some participant scores did not improve to this degree, while others improved more (refer to Table 2 for 95% confidence intervals of the mean difference). Further research should seek to assess for whom this program works most effectively.

Eating disorder-related hospital admissions and costing data related to PMP adds another domain on which to evaluate the program. Fewer hospital admissions were reported during PMP participation, compared to the 6-months prior, which likely represents an important cost saving. We postulate that the intentional peer support offered during the PMP is likely to explain some of this marked decrease at a program level, but further research is required to confirm this and further explore individual variations in response to PMP participation, irrespective of hospital admission. Long hospital waiting lists during COVID-19 may also have had an impact on the lower hospital admission found [42]. This finding offers evidence however that hospital admissions may reduce during PMP, regardless of the broader eligibility criteria for the program from the original pilot which included only people transitioning out of intensive treatment programs [25].

The themes from the qualitative analysis of the PMP feedback questions helped to elucidate mentees experiences of intentional peer support within the current program, thereby improving fidelity of this evaluation. Researchers in the field have acknowledged the importance of using diverse methodology when seeking to understand the complex nature of peer work in recovery [14]. Past in-depth qualitative analysis has been conducted on the initial PMP pilot [26] and main themes were re-confirmed in the current version of the program. Mentees identified the therapeutic nature of empathy, validation, normalisation of experiences and feeling truly seen and understood by another who has ‘walked in their shoes’. The difficult nature of making changes, but the benefit accruing therefrom, highlights the juxtaposition of up and down sides. Similarly, the challenge of mentees allowing themselves to be vulnerable and open in order to benefit as much as possible from the mentor relationship, was also challenged by sadness when the relationship came to an end at program completion. The quantitative and qualitative findings from our current study, taken together, support contemporary dialogues that eating disorder recovery is personal and multi-dimensional, encompassing both the reduction of eating disorder symptoms and building psychological well-being though multiple avenues including fostering meaningful relationships, self-adaptability, resilience and positive personal growth [41]. The current study confirmed outcomes from the initial PMP pilot study [25, 26] and shows efficacy for a broader group of individuals with eating disorder problems, at various stages of their wellness journey. The acceptability of the online delivery was made pressing by the COVID-19 pandemic but has longer-term applicability in expanding availability of the program. We provisionally conclude that peer support during eating disorder recovery can offer significant benefits towards symptomatic improvement, when the quantitative and qualitative data are considered together, and await this being examined in high quality research including embedded lived experience co-production.

Results of the study must be interpreted in light of key limitations. The absence of a control group is a key limitation for understanding whether and to what degree the improvements in symptoms and reductions in hospital admissions might be attributable to non-program effects. This issue should be addressed as a priority for studies moving forward considering a range of contemporary research methodologies and approaches to explore the mechanisms and effectiveness of peer mentoring given the complexities of eating disorder recovery [43]. Furthermore, the impact of prior hospitalisation and limited access to hospital during COVID may both be alternative explanations for our findings. It is also important to note that while average data are presented in this paper, there is inherent variation in the degree of symptom response from individual to individual. We do not yet know characteristics or predictors of whom will benefit significantly from engagement with a peer mentoring program. Other limitations include the potential for selection bias and lack of generalisability. The accuracy of self-reported data and recall bias is also a limitation. Further high quality, co-designed, prospective research utilising a range of study designs and long-term follow up is urgently required on a range of forms of peer support for eating disorder management, to inform implementation into policy and practice.

In conclusion, the findings of this work add to the under-researched but important area of peer mentoring programs in eating disorder management. The current research reports novel findings on the further evaluation of EDV’s PMP for eating disorders, as a matured program, with a larger and more diverse sample, adapted for online delivery. While participating in the program the mentees experienced a significant reduction in eating disorder symptomatology, improved psychological wellbeing and reduced hospital admissions. Thematic analysis showed key benefits experienced by mentees through connecting with people with lived experience of an eating disorder (the mentors), accessing a different kind of space for recovery, building hope, motivation and social engagement, and gaining practical help with the ‘work’ of recovery. Given growing research and anecdotal evidence for the benefits of peer support in eating disorder recovery, and wide-spread government and societal shifts calling for greater lived experience inclusion into mental health programs, targeted research in peer mentoring programs in eating disorder management is likely to have large-scale benefits for society.

### Supplementary Information

**Additional file 1**.

## Data Availability

The data are not publicly available due to privacy.

## Abbreviations

PMP: Peer Mentoring Program
EDV: Eating Disorders Victoria
EDE-Q: Eating Disorder Examination Questionnaire
DASS-21: Depression Anxiety Stress Scale - 21 (also known as Short Form)
