# Supplementary material for: A peer mentoring program for eating disorders: improved symptomatology and reduced hospital admissions, three years and a pandemic on
Source: J Eat Disord. 2024 Jul 15;12:99. doi: 10.1186/s40337-024-01051-7 (PMC11247779; doi:10.1186/s40337-024-01051-7)
Supplement: Supplementary file 1 — Additional file 1. [file 40337_2024_1051_MOESM1_ESM.docx]

**Additional file 1. A Description of the Peer Mentoring Program (PMP).**

A description of EDV’s PMP has been provided in detail elsewhere and is summarised below including key differences from the initial program evaluation (Beveridge et al., 2019; Hanly et al., 2020).

*Mentor training and matching with mentees*

After recruitment, eligibility assessment and PMP enrolment, each mentee was matched with a peer mentor, who were paid employees of EDV specifically for the purpose of undertaking a mentoring role. Mentors are not the focus of this current evaluation, however they were individuals who had recovered from an eating disorder for a minimum of two years duration and acted as peer mentors in the program. All mentors had undergone three days of intensive training and an induction prior to being matched with a mentee. Training included an introduction to Intentional Peer Support Principles (Gillard et al., 2017), a series of self-reflection exercises around setting boundaries, understanding the role of peer support, skills used in mentoring and what is recovery in the eating disorder sector. In addition, mentors completed the internationally recognised safeTALK suicide alertness workshop. EDV also provided all mentors with regular de-briefing and bi-monthly group supervision throughout each of the rounds of the PMP in this study.

Mentees and mentors were paired on the basis of information provided by both parties. Mentees provided information about their preferences for working with a mentor of a same/different: age, gender, eating disorder history, and/or geographic area. Mentors provided information for a ‘Mentor Profile’ which included information on their personal demographics, own eating disorder lived experience, and their values to facilitate effective matching. The final pairings were confirmed by program staff.

*Peer Mentoring Program (PMP) overview*

The PMP involved fortnightly mentoring sessions of up to 3 hours, over 6 months. Mentoring activities were guided by each individualised mentee’s Mentoring Journal formulated during session 1 of the program, which focused on the mentee’s short-term goals in the following domains: living circumstances and skills, health, self-care, social relationships and connectedness, creative interests and hobbies, work/career and education, identity and sense of self, community roles and responsibilities. The Mentoring Journal also outlined the mentee’s current recovery strategies and warning signs that indicate additional professional support or treatment should be sought. Mentors also completed their own Wellness Plan (the ‘Mentor Profile’) which allows them to document self-care strategies and warning signs that may indicate a decline in their own wellbeing.

Sessions 2-12 of the mentoring activities focused on supporting the mentee to work toward their identified goals through community-based activities such as supermarket shopping, cooking, arts, connecting with community or navigating social interactions involving food. Mentors shared aspects of their own recovery stories to promote hope and provide an empathic response to the challenges associated with recovery. Session 13 of the mentoring activities focused on completion of a program summary outlining the mentee’s progress and achievements. This included a future-focused component encouraging the mentee to identify strategies and alternatives supports to draw upon after the program has concluded in order to sustain their progress. An activity budget of approximately $465 per mentor-mentee match, was provided for the 6-months duration of the program.

Throughout the program, mentees were offered a single opportunity to participate in a group session with other participants, facilitated by PMP staff members. These sessions enabled mentees to provide feedback on their experience of the program and increase a sense of community among mentees. Mentors were provided with opportunities to attend bi-monthly group supervision sessions facilitated by PMP staff members, a social worker and a psychologist in order to provide peer to peer support, develop an understanding of their role and increase a sense of community among mentors. As Victoria was in COVID-19 lockdown for the majority of each round evaluated in this study, all activities were largely online.

**References:**

Beveridge, J., Phillipou, A., Jenkins, Z., Newton, R., Brennan, L., Hanly, F., Torrens-Witherow, B., Warren, N., Edwards, K., & Castle, D. (2019). Peer mentoring for eating disorders: results from the evaluation of a pilot program. *Journal of Eating Disorders*, *7*. <https://doi.org/http://dx.doi.org/10.1186/s40337-019-0245-3>

Gillard, S., Foster, R., Gibson, S., Goldsmith, L., Marks, J., & White, S. (2017). Describing a principles-based approach to developing and evaluating peer worker roles as peer support moves into mainstream mental health services. *Mental Health and Social Inclusion*, *21*(3), 133-143.

Hanly, F., Torrens-Witherow, B., Warren, N., Castle, D., Phillipou, A., Beveridge, J., Jenkins, Z., Newton, R., & Brennan, L. (2020). Peer mentoring for individuals with an eating disorder: a qualitative evaluation of a pilot program. *Journal of Eating Disorders*, *8*(1), 29. <https://doi.org/10.1186/s40337-020-00301-8>
